# Early angiography improves postoperative clinical outcomes compared to delayed angiography among patients with vascular pathologies following partial nephrectomy

**DOI:** 10.1007/s00345-025-05491-x

**Published:** 2025-03-18

**Authors:** Rinat Lasmanovich, Husny Mahmud, Boris Khaitovich, Dorit E. Zilberman, Barak Rosenzweig, Menachem Laufer, Orith Portnoy, Avi Epstein, Avinoah Irony, Zohar A. Dotan

**Affiliations:** 1 Department of Urology, Sheba Medical Center, Tel-Hashomer, Israel, Tel-Aviv University, Tel-Aviv, Israel; 2 Unit of Interventional Radiology, Sheba Medical Center, Tel-Hashomer, Israel, Tel-Aviv University, Tel-Aviv, Israel; 3 Department of Diagnostic Imaging, Sheba Medical Center, Tel-Hashomer, Israel, Tel-Aviv University, Tel-Aviv, Israel; 4 Department of Emergency Medicine, Sheba Medical Center, Tel-Hashomer, Israel, Tel-Aviv University, Tel-Aviv, Israel

**Keywords:** Hematuria, Partial nephrectomy (PNx), Renal artery pseudoaneurysm (RAP)

## Abstract

**Purpose:**

This study aims to assess the evaluation, management, clinical outcomes and incidence of postoperative hematuria following partial nephrectomy (PNx) for renal tumors.

**Methods:**

We retrospectively reviewed the medical charts of 936 adult patients who underwent PNx between 2008 and 2023. Patients presenting with hematuria during the first 6 months of surgery were included. Group 1, comprising patients who were treated with early angiography and selective embolization (*n* = 8), was compared to Group 2, patients who underwent imaging first (US or CTA), followed by angiography and selective embolization (*n* = 10, “delayed” angiography).

**Results:**

24 (2.6%) patients presented with hematuria, 18 (75%) required angiography-assisted intervention. Of those 18 patients, 17 (94.4%) were diagnosed with vascular pathologies; renal artery pseudoaneurysm (RAP) and arteriovenous fistula. Ultrasound (US) did not detect RAP in 33% of patients’ initial evaluations (67% sensitivity). The median age was 67 years (IQR: 71.5–58.5 years), and the median time to hematuria was 11.5 days (IQR: 20.3- 7 days). The difference in the median interval time from presentation to embolization between Groups 1 and 2 was 20.2 h (CI 95%, *p* = 0.25). Group 1 had higher hemoglobin levels following therapy (*p* = 0.04), lower transfusion rates or antibiotic therapy (*p* = 0.02), shorter hospitalization stays (*p* = 0.03), and lower re-admission rates (*p* = 0.043*)* compared to Group 2.

**Conclusion:**

RAP is ubiquitous among patients presenting with hematuria following PNx. With hematuria presentation, the use of US should be limited. For cases where selective embolization is considered, angiography is sufficient to identify vascular pathologies, guiding therapeutic intervention. Management by early angiographic intervention is associated with better clinical outcomes compared to delayed angiography following confirmatory imaging.

**Supplementary Information:**

The online version contains supplementary material available at 10.1007/s00345-025-05491-x.

## Introduction

Delayed hematuria following partial nephrectomy (PNx) is uncommon and most frequently caused by a renal artery pseudoaneurysm (RAP). RAP is a rare vascular complication resulting from renal surgery, percutaneous procedures, renal biopsy, penetrating trauma, and, more infrequently, following blunt trauma [1–3]. It can manifest as macro hematuria, flank pain, syncope, or fever, and may occasionally lead to hemodynamic instability and potentially life-threatening states [5, 6]. The incidence of RAP following PNx reported in the literature ranges between 0.43 and 5% [2, 3, 7, 8] within a median time to presentation ranging between 12 and 14 days [1–11]. Its uncommon occurrence calls for a high level of clinical awareness.

There is no standard assessment for evaluating postoperative hematuria after PNx [10, 12]. Imaging options include ultrasonography (US), computed tomographic angiography (CTA), and renal artery angiography. Color US Doppler is a useful, easy, and cost-effective method, although it has several limitations. For example, dependency upon operator experience and the possibility of being affected by body habitus. CTA is highly accurate, with a specificity of 90-95.1% and sensitivity of 98.7-100% for detecting RAP [11, 13]. Angiography is an invasive procedure and although it is highly diagnostic and therapeutic, it is associated with radiation exposure and invasiveness that are unnecessary in non-RAP cases.

This study aims to evaluate the initial diagnostic process including the imaging methodology of choice as well as subsequent therapy for patients who had gross hematuria between 1 day and 6 months following PNx.

## Patients and methods

### Study design and setting

We retrospectively reviewed the medical records of 936 adult patients who underwent robotic, laparoscopic, or open PNx in our institution between 2008–2023. Using big data software (MDClone, ADAMS Platform, Israel), we extracted data on patients diagnosed at the emergency department with hematuria during the post-operation period. We refer to hematuria as the presence of blood in urine with or without loin pain. The key words that were used during the identification process were “hematuria”, “hematuria syndrome”, “gross” or ‘macroscopic hematuria”. Time frames were set at 1 day to 6 months following the operation date.

### Selection of participants

**Fig. 1:**
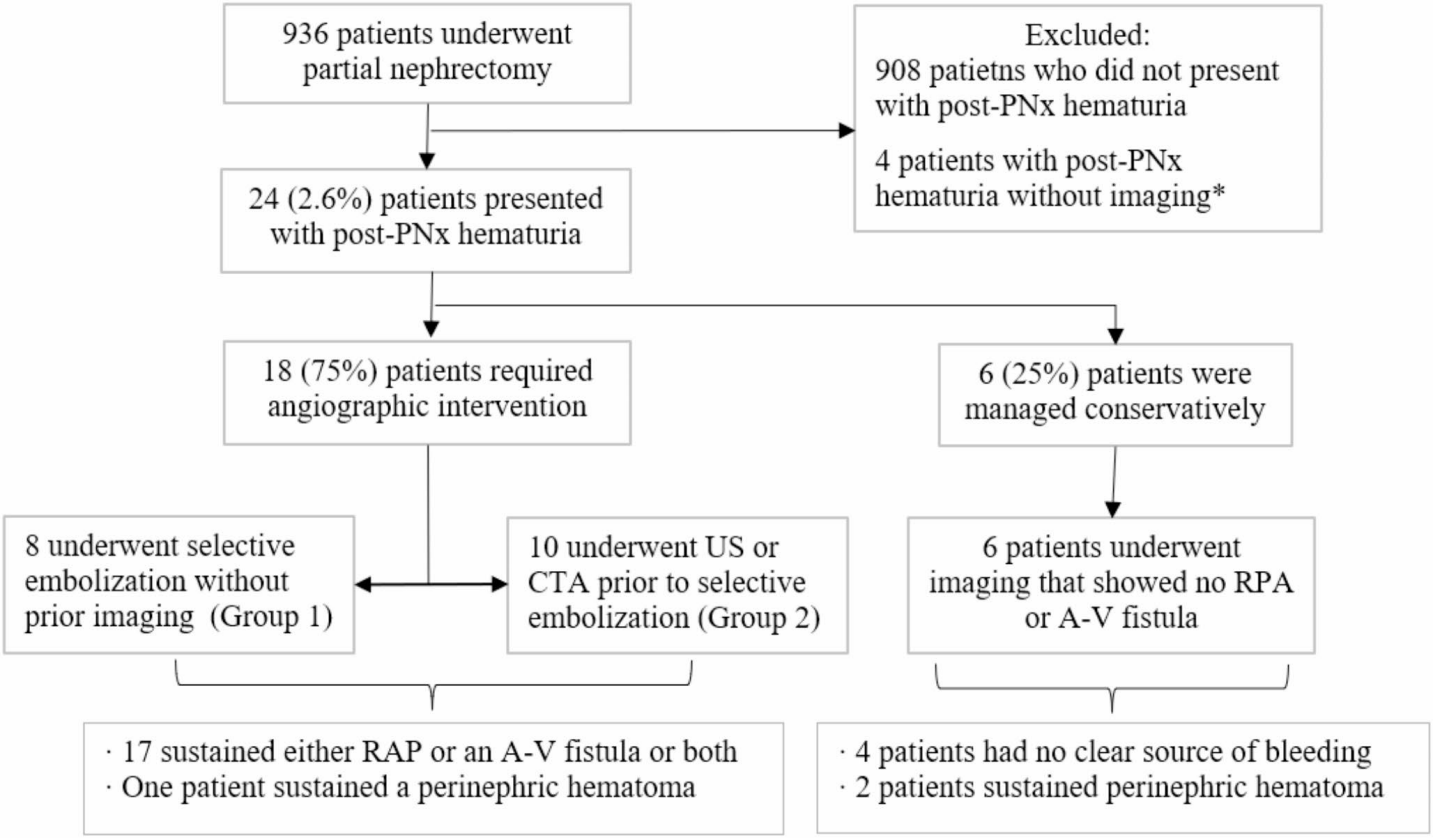
Patient selection, imaging modality and final diagnosis. *4 patients did not undergo US\CTA nor angiography with selective embolization. PNx: partial nephrectomy, CTA: computed tomography angiography, US: ultrasound, RAP: renal artery pseudoaneurysm, AV: arteriovenous

Patients diagnosed with hematuria 1 day to 6 months following PNx and had undergone imaging studies (US or CTA) during the diagnostic evaluation and\or underwent angiographic intervention were included in the study. Demographic characteristics (age, sex, medical history), tumor characteristics (side, size, nephrometry score), index procedure (surgical approach, estimated blood loss [EBL], warm ischemia length), laboratory results, radiographic, angiographic findings, and outcome following therapy for hematuria were collected. Patient selection is summarized in Fig. 1.

The total cohort was comprised of two groups: Group 1 included patients with hematuria who were treated by definitive treatment e.g., arterial angiography and selective embolization (“early” arterial angiography); Group 2 included patients with hematuria who underwent imaging (US or CTA) before definitive treatment (“delayed” arterial angiography). Hemodynamically unstable patients were included in both groups.

### Outcomes

The primary outcomes were, time from presentation to definitive treatment, accuracy of imaging methods used during the evaluation process, and RAP incidence at diagnosis.

The secondary outcomes were hemoglobin (Hg) levels, estimated glomerular filtration rate (eGFR) and △eGFR (was defined as the post-PNx eGFR value minus eGFR following hematuria). Regarding CTA, the null hypothesis was that CTA followed by angiography does not cause greater renal impairment compared to angiography alone. Additional clinical parameters such as blood transfusions, antibiotic treatment, hospitalization length and re-admission rates were also assessed.

### Ethics

The study was performed under the ethical standards of the Declaration of Helsinki and its later amendments. The protocol was approved by the Ethics Committee of Sheba Medical Center (approval SMC-4146, approval date: 29.1.2007). We treated the data according to the principles of good clinical practice (GCP).

### Statistical analysis

Continuous variables that followed a normal distribution were shown as means with standard deviations (SDs) in the descriptive statistical analysis, while variables that did not follow a normal distribution were shown as medians with interquartile ranges (IQRs). The frequencies and percentages of categorical variables were used. The independent sample t-test for normally distributed data was used to compare continuous variables between groups. The Mann-Whitney U test was used for abnormally distributed data. The chi-squared test was applied to evaluate categorical variables. Statistical significance was determined at a p-value of less than 0.05. All statistical analyses were performed using IBM SPSS software (version 29).

## Results

### Cohort description

Of 936 adult patients who underwent minimally invasive or open PNx in our institution between 2008 and 2023, 24 (2.6%) presented with hematuria following PNx, 18 (75%) required angiographic intervention as they showed no improvement under conservative treatment. Of those, 17 patients needed embolization due to vascular pathologies, and one due to perinephric hematoma. The median age was 67 years (IQR:71.5–58.5), and the median time to the hematuria presentation following PNx was 11.5 days (IQR:20.25-7). The median tumor size was 4 cm (IQR:5-3.13). The surgical approach was open in 13 procedures, laparoscopic in 4, and robotic in 7. The mean EBL was 125 ml (*±* SD 243.5), with a mean warm ischemic time of 22 min (*±* SD 6.05). Patient and tumor characteristics are summarized in Tables 1 and 2.

**Table 1:** Patient demographic, tumoral and surgical characteristics

Variables	Patients presenting with hematuria (*n*=24)	Group 1 - Immediate angiography (*n*=8)	Group 2 – Delayed angiography* (*n*=10)	*P* value
Age, years, median (IQR)	67 (71.5-58.5)	69 (70-59.25)	64 (70.75-56)	0.6
Male, n (%)	19, (79.2%)	6 (75%)	8 (80%)	0.8
Preoperative medical history				
HTN, n (%)	18 (75%)	7 (88%)	8 (80%)	0.67
DLP, n (%)	15 (63%)	4 (50%)	7 (70%)	0.38
IHD, n (%)	6 (25%)	2 (25%)	4 (40%)	0.5
DM, n (%)	8 (33.3%)	3 (38%)	5 (50%)	0.91
Tumor size (cm) median (IQR)	4 (IQR:5-3.13)	4 (4.85-3.75)	4.2 (5.38-4)	0.4
Left-sided tumor	12 (50%)	5 (63%)	4 (40%)	0.4
Surgical approach				0.625
OPN, n (%)	13 (54%)	4 (50%)	7 (70%)	
LPN, n (%)	4 (16.7%)	2 (25%)	1 (10%)	
RPN, n (%)	7 (29%)	2 (25%)	2 (20%)	
EBL, mL, mean (±^±^SD)	125 (±243.5)	230 (±198)	173 (±191.53)	0.6
Warm ischemic time (min), mean (±SD)	22 (±6.05)	19.67 (±6.62)	25 (±4.12)	0.07
R.E.N.A.L Nephrometry score, median (IQR)		6 (9-5.5)	8 (10-6)	0.28
Postop. Cr (mg/dl), median (IQR)	1.2 (1.51-1.1)	1.18 (1.22-1.12)	1.2 (1.59-1.08)	0.72
Postop. Hg (g/dL), mean (±SD)	10.92 (±1.57)	11.84 (±1.53)	10.9 (±1.72)	0.24
Post-PNx eGFR mL/min/1.73m^2^, median (IQR)	66.09 (71.13-49.44)	66.73 (70.49-61.51)	68.84 (77.57- 49.67)	0.72

*Embolization from any reason (including perinephric hematoma, *n* = 1)

SD: standard deviation, n: number, HTN: hypertension, DLP: dyslipidemia, IHD: ischemic heart disease, DM: diabetes mellitus, IQR: interquartile range, OPN: open partial nephrectomy, LPN: laparoscopic partial nephrectomy, RPN: robotic partial nephrectomy, EBL: estimated blood lose, min: minutes, Postop: postoperative, Cr: creatinine, Hg: hemoglobin, eGFR: estimated glomerular filtration rate. mL: milliliter

**Table 2:** Nephrostomy scores and pathology for Group 1 and Group 2 patients

Patients	Group No.	Gender*	Age	*R*	E	*N*	A	L	Renal Nephrometry Score	Pathology
1	1	1	69	2	3	3	1	2	10a	T1b LG RCC
2	1	0	60	1	1	3	1	1	6a	T1a RCC
3	1	0	47	1	3	3	2	2	9p	T1a papillary RCC
4	1	0	73	1	1	1	1	1	4a	^
5	1	0	69	2	1	2	2	1	6p	T1B G2 RCC
6	1	0	76	1	1	1	3	1	4x	T1a G2 RCC
7	1	1	57	3	1	3	3	2	9x	Cystic nephroma
8	1	0	69	2	1	2	1	1	6a	T1b Sarcomatoid G3 RCC
9	2	1	64	1	1	1	1	1	4a	T1a RCC
10	2	0	73	1	2	1	3	2	6x	Oncocytoma
11	2	0	47	2	1	3	1	2	8a	T1a RCC
12	2	0	65	1	3	3	1	3	10a	T1b RCC
13	2	0	71	1	2	1	2	2	6p	T1a chromophobe
14	2	0	59	2	2	2	3	2	8x	T1b RCC
15	2	0	70	1	3	3	2	3	10ph	Oncocytoma
16	2	0	71	2	1	1	1	2	6a	T1b RCC
17	2	0	55	2	3	3	1	5	11a	T1b RCC
18	2	1	41	3	2	3	3	5	11a	T1b RCC

* “0” represents male patients, ”1” represents female patients

^ Data is missing

R.E.N.A.L Score. Radius; Largest diameter in any single plane (cm), Exophytic/endophytic%; mass exophytic vs. endophytic relative to renal parenchyma, Nearness to collecting system or sinus (mm), Anterior/posterior (“a”:anterior, “p”:posterior, “x”: neither); Primary location of tumor relative to coronal plane at level of hilar vessels, Location relative to polar lines, Hilar tumor (h)

L; low, G; grade, RCC; renal cell carcinoma

## Main results

The median interval time from ED presentation to embolization was 13 h (IQR: 6.8–48.2) and 33.2 h (IQR; 15.7–96) for the patients in Group 1 and Group 2, respectively (CI 95%, *p* = 0.25).

**Fig. 2:**
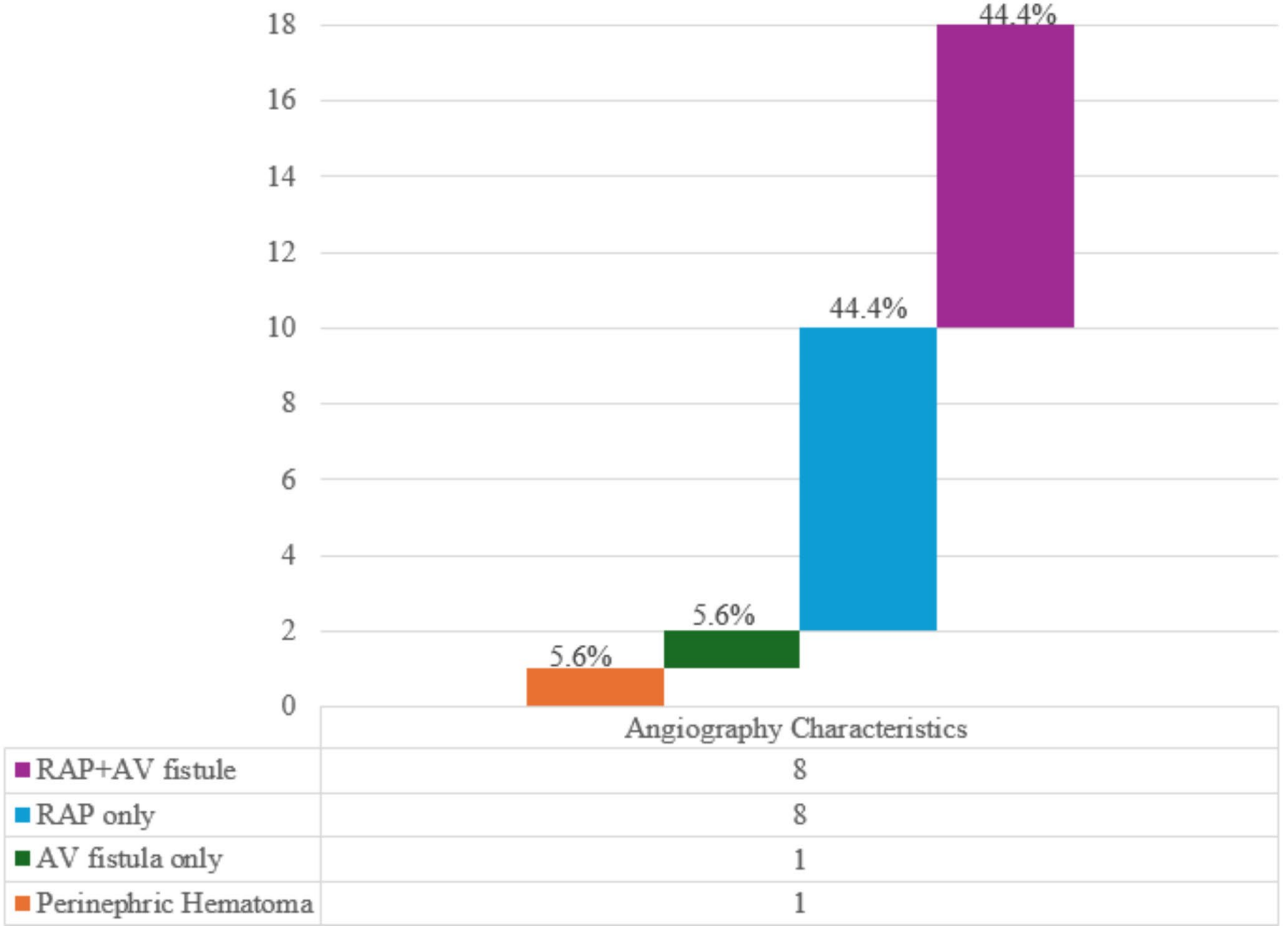
Angiographic Characteristics Column. Describing angiographic features in patients with hematuria following PNx required an angiography intervention. RAP only – 8 cases, 44.4% (purple), RAP with A-V fistula 8 cases, 44.4% (light blue), A-V fistula only – 1 case, 5.6% (green), Perinephric hematoma − 1 case, 5.6% (orange)

Radiographic and angiographic features were collected to characterize the size and incidence of RAP, which was found in 16 patients (66.7%); RAP incidence was 7 (87.5%) for Group 1 and 9 (90%) for Group 2. Among Group 2, 6 patients underwent US and 4 underwent CTA. While 4\4 CTA corresponded with angiography reports, 2\6 US tests did not (showed no evidence of RAP while in fact RAP existed). Based upon the 4 CTA tests, the median RAP size was 15 mm (IQR: 8–18). All definitive treatment procedures were performed successfully, and none required surgical exploration or nephrectomy. Angiographic features are described in Fig. 2.

During hospitalization (defined as the period from admission after ED presentation, until definitive treatment), Hg levels were significantly lower in Group 2 compared to Group 1 (9.06 *±* 0.9 ml\dl vs. 10.43 *±* 1.98 ml\dl, respectively, P *=* 0.04). More patients from Group 2 required blood transfusions or antibiotic therapy (8 patients [80%] vs. 2 patients [25%] CI 95%, *P* = 0.02). Patients from Group 2 had higher blood transfusion rates during hospitalization (7 blood transfusions vs. 1, CI 95%, *P* = 0.2), and longer provision of antibiotic therapy (a cumulative value of 33 days of treatment vs. 5 days in Group 1, CI 95%, *P* = 0.37).

Total hospitalization stay was significantly lower among Group 1 (5.64 *±* 3.22 vs. 9.5 *±* 4.28 days, CI 95%, *P* = 0.03). In addition, there was a higher rate of re-admission among Group 2 patients (4 [40%] vs. 0 [0%], CI 95%, *P* = 0.043). No significant group differences were found in △eGFR (18.95 *±* 20.3 for Group 1 vs. 8.96 *±* 9.5 for Group 2, CI 95%, *P* = 0.19). Timeframes and laboratory evaluation findings during the episodes of post-PNx hematuria are summarized in Table 3. Data on eGFR analysis is summarized in *supplementary information*.

**Table 3:** Timeframes and laboratory evaluation findings during the episodes of post-PNx hematuria

Variables	Patients presenting with hematuria (*n* = 24)	Group 1 - Immediate angiography (*n* = 8)	Group 2 – Delayed angiography* (*n* = 10)	*P* value
Days from surgery to ED presentation, mean (*±* SD)	11.5 (*±* 9.04)	9 (*±* 5.63)	17.4 (*±* 7.12)	**0.015**
Anti-aggregate therapy during hematuria, *n* = 21 (%) ^**^**^	3 (12.5%)	0 (0%)	2 (22.2%)	0.33
Hg (g\dL) at ED presentation, mean (*±* SD)	10.74 (*±* 1.58)	11.42 (*±* 1.8)	10.74 (*±* 1.31)	0.38
Cr (mg/dl) at ED presentation, median (IQR)	1.26 (1.62–1.1)	1.23 (1.46–1.09)	1.27 (1.92–1.1)	0.66
Hemodynamic instability at ER presentation, n (%)	2 (8.3%)	1 (12%)	1(10%)	0.87
Time from ED to embolization, hours (IQR)	14 (96 − 7.5)	13 (48.25–6.85)	33.25 (96-15.8)	0.25
Need for 2nd embolization, n (%)	3 (12.5%)	2 (25%)	1 (10%)	0.39
Need for blood transfusion or Abx, n (%)	13 (54.2%)	2(25%)	8(80%)	**0.02**
Days of Abx use, median (IQR)	43 (3 − 0)	5 (0,0)	33 (1.5, 6 − 0)	0.14
Lowest Hg (g/dL) during 2nd hospitalization, mean (*±* SD)	9.53 (*±* 1.58)	10.43 (*±* 1.98)	9.06 (*±* 0.9)	**0.04**
Hg (g/dL) at discharge mean (*±* SD)	10.5 (*±* 1.29)	11.08 (*±* 1.44)	10.22 (*±* 0.87)	0.16
Highest Cr (mg/dL) level during 2nd hospitalization, median (IQR)	1.54(1.79–1.26)	1.54 (1.78–1.28)	1.29 (1.76–1.17)	0.7
eGFR post-PNx hematuria mL/min/1.73m^2^, mean (*±* SD)	49.47 (*±* 18.71)	45.03 (*±* 14.86)	51.96 (*±* 21.89)	0.47
Length of stay, total days, mean (*±* SD)	5 (*±* 5.87)	5.64 (*±* 3.22)	9.5 (*±* 4.28)	**0.03**
More than one hospitalization required, n	4 (16.6%)	0 (0%)	4 (40%)	**0.043**

^**^**^For patients whose data were available

IQR: interquartile range, ED: emergency department, Hg: hemoglobin, Cr: creatinine, n: number, ABx: antibiotics. eGFR: estimated glomerular filtration rate, mg: milligram, g: gram, mL: milliliter, dL; deciliter, min: minute

**Bold** denotes significant

The group of patients that presented with hematuria but had no diagnosed vascular pathology and therefore needed no angiographic intervention, were treated conservatively (*n* = 6). All 6 patients were stable and had higher Hg levels at presentation compared to post-surgery discharge. 5 underwent abdominal CT (2 were diagnosed with perinephric hematoma), and 1 patient underwent US (normal findings). In these 6 patients, hematuria improved with bladder irrigation, except for 2 patients that needed blood transfusion. Their median hospitalization time was 4.5 days, without re-admissions or complications.

## Discussion

The occurrence of gross hematuria after PNx is uncommon, with RAP and AV fistula being the most prevalent causes. The incidence of RAP reported in the literature during the last decade varies and ranges between 0.43 and 5% according to the surgical approach and tumor location [2, 3, 7, 8, 14]. A recent publication demonstrated that robotic PNx is associated with a lower risk of RAP compared to open and laparoscopic PNx [15]. Gross hematuria is the presenting symptom of RAP in 100% of the cases [16, 15, 17], while other symptoms such as fever, flank pain, hypotension, bloody discharge from the drain site, dizziness, and syncope may also appear [18, 19, 17]. Our findings were similar; hematuria following PNx presented in 24 patients (2.6%), of them 18 needed angiographic intervention, of whom 17 were diagnosed with vascular pathologies (94.4%). RAP was found in 88.9% of all cases of vascular pathology that required intervention.

Currently, literature lacks a standard or uniform assessment for diagnosing RAP after PNx [10, 12]. The options include early angiography or other imaging modalities, such as US and CTA. CTA is considered the diagnostic study of choice for the evaluation of hematuria following PNx among hemodynamically stable patients [6, 13, 19, 17]. In the current study, RAP was not detected by US in 33% of the patients in their initial evaluation at the ED (67% sensitivity).

Sonography is considered a less sensitive technique compared to CTA in the diagnosis of vascular pathology. Katyel et al. showed that US Doppler failed to diagnose vascular pathologies, including pseudoaneurysm [20]. Mustafa et al. demonstrated that despite reports of sensitivity and specificity of 94% and 97%, respectively, in the diagnosis of post-catheterization pseudoaneurysms in upper and lower extremities (i.e., femoral and radial arteries), sonography has low sensitivity in the evaluation of deep visceral artery pseudoaneurysms (duodenum, pancreas, and renal arteries) [21]. Although US may limit the amount of radiation and intravenous contrast medium (CM) exposure, its low-resolution images may lead to equivocal or false-negative results [22]. Thus, the use of US for detecting RAP after PNx should be limited.

In contrast, CTA is not dependent upon the technologist or the patient’s habitus [21, 20]. A multicenter study demonstrated that CTA was the most used modality for the diagnosis and evaluation of renal artery aneurysms (58%) [23]. According to the Society for Vascular Surgery, CTA is considered the diagnostic study of choice for renal aneurysms (B1 recommendation) [24]. Accordingly, CTA did not produce false negative results, with five CTA tests aligned with angiography reports for vascular pathologies and perinephric hematomas. This finding supports the notion that CTA should be performed as the modality of choice to efficiently diagnose RAP.

Despite the high sensitivity and specificity of CTA, the use of CM has the potential to impair renal parenchyma and subsequently reduce kidney function. In this cohort, a significant decline in eGFR was observed post-PNx hematuria across both groups, with Group 2 exhibiting a more pronounced reduction. Patients underwent angiographic intervention displayed elevated △eGFR levels, though intergroup differences remained statistically insignificant, even upon subgroup analysis. This may be explained by using CM during CTA, or by the angiography performance (renal damage by kidney embolization and the use of a CM) or both. As we explore the impact of CTA on renal function, we found that △eGFR was not significantly different between the groups, nor in sub-group analysis for patients who undergo both CTA and angiographic intervention. Nevertheless, given the small sample size of the subgroup (*n* = 4) and the higher initial eGFR values in Group 2, the cumulative renal insult from repeated CM administration warrants consideration. Hence, in our view, when selective embolization is considered, performing CTA may not be necessary, as angiography sufficiently identifies and localizes vascular pathologies, guiding therapeutic intervention. With the data at hand, we did not reject the null hypothesis.

Group 2 patients received definitive treatment later than Group 1. Although time differences were not significantly different, clinical outcomes between the groups cannot be overlooked, since a shorter waiting time for definitive treatment demonstrated several advantages, including higher Hg levels during hospitalization (assumably due to bleeding control), lower rates of blood transfusion and antibiotic use, shorter hospitalization time and lower re-admissions. These findings suggest that early embolization treatment for post-PNx hematuria may lead to; (1) reduced likelihood of morbidity and mortality which are associated with low Hg levels, especially in patient with comorbidities and increased age [25–26]; (2) a reduction in potential side effects following unnecessary use of blood products; (3) reduced hospital financial expenses, due to shorter hospitalization and reduced re-admission. Moreover, an early angiography is associated with better clinical outcomes due to prompt cessation of the bleeding episode even for stable patients (91.7% of our cohort). Finally, there were no significant group differences in demographic data, tumoral or surgical characteristics, vital signs and Hg levels at presentation, indicating comparable bleeding severity. 

Hemorrhagic complications post-PNx-associated hematuria may be life-threatening and should be treated promptly [14, 16, 27]. Conservative management may be appropriate for patients with less severe symptoms, including close observation, absolute bed rest, serial Hg measurements, catheter insertion and bladder irrigation, and blood transfusion when needed [28]. Selective angiography and therapeutic coil embolization of renal artery pseudoaneurysms are indicated in the presence of decreased Hg levels, unresponsiveness to blood transfusion, and hemodynamic instability [29, 17]. Both groups consist hemodynamically unstable patients suggesting low probability of bias: In Group 1 the patient had significant comorbidities and a low Hg level (7.2ng\ml) at presentation compared to Group 2 (8.5ng\ml), who was stabilized following conservative treatment and had embolization 144 h from presentation since hematuria persisted (US was performed at the request of the angiography unit).

Our study has several limitations, beginning with its retrospective design. Since the data were collected from a single tertiary center, generalizability may not apply to all populations, even though our study population is heterogenic coming from diverse geographic and socioeconomic areas nationwide. Secondly, the paper includes all the patients who underwent robotic, laparoscopic and open surgeries, which introduces selection bias. In addition, the time to embolization was relatively high in both groups, with significant overlap, making it challenging to draw unequivocal conclusions. Moreover, although we viewed a large sample size of 936 patients, the final analysis was performed on a subgroup consisting of 18 patients. That said, we explored a rare complication following PNx, thus there is a low likelihood of performing a randomized trial on angiography timing for post-PNx hematuria.

## Conclusions

The overall incidence of vascular pathologies in hematuria presenting up to 6 months from PNx reached more than two-third of patients, as RAP being the most common etiology. In post-PNx hematuria, the use of US should be limited. For cases where selective embolization is considered, angiography is sufficient to identify and localize vascular pathologies, guiding therapeutic intervention. On this matter, with the data at hand, we did not reject the null hypothesis.

Early angiographic intervention is associated with higher Hg levels, short hospital stays, and lower re-admission rates compared to delayed angiography due to confirmatory imaging (US or CTA). Early angiographic intervention was free of procedure-related complications. Hence, we recommend early angiography as the preferred therapy for patients presenting with post-PNx hematuria.

Further studies are needed to establish hemoglobin thresholds, renal function parameters, and optimal timing for embolization in stable patients that may influence clinical outcomes.

## Electronic supplementary material

Below is the link to the electronic supplementary material.

Supplementary Material 1

## Data Availability

No datasets were generated or analysed during the current study.
