# Supplementary material for: Early angiography improves postoperative clinical outcomes compared to delayed angiography among patients with vascular pathologies following partial nephrectomy
Source: World J Urol. 2025 Mar 18;43(1):177. doi: 10.1007/s00345-025-05491-x (PMC11920357; doi:10.1007/s00345-025-05491-x)
Supplement: Supplementary file 1 — Supplementary Material 1 [file 345_2025_5491_MOESM1_ESM.docx]

| Parameters | Comparison between groups |  | P value |
| --- | --- | --- | --- |
|  | Pre-Group 1 | Post-Group1 | 0.033 |
| eGFR | Pre-Group2 | Post-Group2 | 0.015 |
|  | Pre-hematuria patients^ | Post-hematuria patients^ | 0.81 |
| Sub-group analysis | Pre_4CTA* | Post_4CTA* | 0.076 |
| △eGFR** | △eGFR_hematuria patients^ | △eGFR_Group1 | 0.09 |
|  |  | △eGFR_Group2 | 0.08 |
|  |  | △eGFR_Group 1+2 | 0.01 |
|  | △eGFR_Group1 | △eGFR_Group2 | 0.23 |
| Sub-group analysis | △eGFR_Group1 | △eGFR_4CTA* | 0.67 |

^Patients who presented with hematuria, which did not categorize into Group 1 and Group 2 (i.e., did not undergo angiographic intervention)

*Patients that underwent CTA and angiographic intervention, *n*=4

**△eGFR= (post-PNx eGFR value)- (eGFR value following hematuria)
